# Novel Tetrazole-Based Antimicrobial Agents Targeting Clinical Bacteria Strains: Exploring the Inhibition of *Staphylococcus aureus* DNA Topoisomerase IV and Gyrase

**DOI:** 10.3390/ijms23010378

**Published:** 2021-12-29

**Authors:** Piotr Roszkowski, Jolanta Szymańska-Majchrzak, Michał Koliński, Sebastian Kmiecik, Małgorzata Wrzosek, Marta Struga, Daniel Szulczyk

**Affiliations:** 1 Faculty of Chemistry, University of Warsaw, Pasteura 1, 02-093 Warszawa, Poland; roszkowski@chem.uw.edu.pl; 2 Chair and Department of Biochemistry, Medical University of Warsaw, 02-097 Warszawa, Poland; jolanta.szymanska@wum.edu.pl (J.S.-M.); marta.struga@wum.edu.pl (M.S.); 3 Bioinformatics Laboratory, Mossakowski Medical Research Institute, Polish Academy of Sciences, 5 Pawinskiego St., 02-106 Warsaw, Poland; mkolinski@imdik.pan.pl; 4 Biological and Chemical Research Centre, Faculty of Chemistry, University of Warsaw, 02-089 Warsaw, Poland; sekmi@chem.uw.edu.pl; 5 Department of Biochemistry and Pharmacogenomics, Faculty of Pharmacy, Medical University of Warsaw, 02-097 Warsaw, Poland; malgorzata.wrzosek@wum.edu.pl

**Keywords:** antimicrobial, antibacterial, tetrazole, gyrase, topoisomerase

## Abstract

Eleven novel imide-tetrazoles were synthesized. In the initial stage of research, in silico structure-based pharmacological prediction was conducted. All compounds were screened for antimicrobial activity using standard and clinical strains. Within the studied group, compounds **1**–**3** were recognized as leading structures with the most promising results in antimicrobial studies. Minimal inhibitory concentration values for compounds **1**, **2**, **3** were within the range of 0.8–3.2 μg/mL for standard and clinical Gram-positive and Gram-negative bacterial strains, showing in some cases higher activity than the reference Ciprofloxacin. Additionally, all three inhibited the growth of all clinical *Staphylococci* panels: *Staphylococcus aureus* (T5592; T5591) and *Staphylococcus epidermidis* (5253; 4243) with MIC values of 0.8 μg/mL. Selected compounds were examined in topoisomerase IV decatenation assay and DNA gyrase supercoiling assay, followed by suitable molecular docking studies to explore the possible binding modes. In summary, the presented transition from substrate imide-thioureas to imide-tetrazole derivatives resulted in significant increase of antimicrobial properties. The compounds **1**–**3** proposed here provide a promising basis for further exploration towards novel antimicrobial drug candidates.

## 1. Introduction

Antibiotic resistance is mentioned as one of the most important health threats of present times. Increasing appearance of multi-drug-resistant organisms outside the hospital environment confirms their presence in our everyday life. The situation is worsening due to the lack of effective new antimicrobial molecules, and the inappropriate use of available antibiotics. Clinicians have limited appropriate therapeutic options for infected patients [1].

We can divide resistance mechanisms into two general categories, internal and acquired. Internal resistance mechanisms are most often associated with chromosomal coding. Such mechanisms include non-specific efflux pumps, enzymes that block action of antibacterial substances, and other mechanisms that are responsible for the permeability reduction of the antibiotic. The core genetic structure of the bacteria organism is responsible for the creation of these mechanisms. Typically, low level antibiotic resistance is observed when an internal mechanism dominates. However, in the population of immunocompromised patients, pathogens containing internal mechanisms can develop strong antibiotic resistance. Contrariwise, acquired resistance arises as a result of horizontal gene transfer (HGT), involving the process of plasmid-encoding specific efflux pumps and enzymes that are able to modify the antibiotic or its target. This type of resistance mechanism poses a much greater threat to bacteria-infected individuals. We can also use an alternative classification of antimicrobial resistance mechanisms, divided into: active drug efflux, drug inactivation, drug target modification, and drug absorption reduction [2,3,4,5,6,7,8,9,10].

The increase of morbidity and mortality is directly related to the limited number of therapeutic options that can be used by the patients struggling with antibiotic-resistant infections. Consecutively, this causes the need for longer treatments and possible extended hospitalizations, which are also linked with an increased total cost of patient recovery. Methicillin-resistant *Staphylococcus* *aureus* infections might be our greatest concern. The mortality caused by these strains remains at the level of approximately 20–40% [11,12,13]. *Staphylococci* have developed very effective resistance mechanisms against the currently used antibiotics. Examples are shown in Table 1 [14]. The great need for development of new molecules active against these bacterial strains is undeniable.

Details on mechanisms of bacteria strains resistance have been published in many papers [15,16,17,18,19,20,21] and further documents will be released in the future, since new outcomes will be observed. A common assumption from most of the publications is that there is a strong need for new antibacterial compounds that may be valuable in the treatment of bacterial infections resistant to commonly used antibiotics.

In previous years, our research group was devoted to searching for new tetrazole-based compounds with antimicrobial activity [22,23,24,25]. Clinicians use the tetrazole-derived antimicrobial drugs such as Cefamandole, Ceftezole, both second-generation broad-spectrum cephalosporin antibiotics, and the oxazolidinone-class antibiotic Tadalizolid. Tetrazole structural modification of compounds may increase antibacterial properties, which was observed in our studies. In this work, we decided to look for compounds with well-known activity against bacterial strains in our own compounds library. Based on earlier study results and currently developed research we have decided to design imide-tetrazole hybrid structures for antimicrobial evaluation. Our team is investigating a number of imides and their derivatives, reporting diverse structural and biological activities [26,27,28]. Thiourea derivatives of two imides with confirmed antimicrobial activity were used as substrates for the synthesis of tetrazole-imide hybrids [29,30]. Our previous studies showed that the introduction of tetrazole arrangement in thiourea derivatives leads to an increase of antimicrobial activity [22,23,24,25].

## 2. Results and Discussion

### 2.1. In Silico Structure-Based Pharmacological Prediction

#### 2.1.1. Antibacterial Activity

Using AntiBac-Pred [31] web services of Way2Drug platform, activity against Gram-positive and Gram-negative bacteria was predicted for eleven synthesized compounds. It was found that the whole group might be active against *S. aureus*, *Bacillus subtilis*, and *Mycobacterium smegmatis* strains. It should be mentioned, that interesting probability scores were found for compounds:

- **3** (confidence value 0.1880), **9** (confidence value 0.2211), and **12** (confidence value 0.1718) for activity against *Staphylococcus aureus* subsp. *aureus MW2* resistant strain.
- **2** (confidence value 0.1431) and **7** (confidence value 0.2540) for activity against *Bacillus subtilis* subsp. *subtilis str. 168* strain.
- **2** (confidence value 0.1158), **7** (confidence value 0.1011), and **9** (confidence value 0.1511) for activity against *Mycobacterium smegmatis* strain.

Probability scores should be considered as low, but the whole group of designed compounds showed potential activity against similar strains.

#### 2.1.2. Toxicological Parameters

The pkCSM [32] software was used for evaluation of toxicological parameters in silico. Evaluation revealed that two of the synthesized compounds were positive in the AMES test, therefore should be recognized as carcinogenic. It should be mentioned that both contained -NO_2_ substituent attached to phenyl ring. It is commonly known that this moiety may lead to compounds with high toxicity. Furthermore, all derivatives except **10** were found to have the human Ether-à-go-go-Related Gene (hERG II) inhibitor, which is the principal cause of acquiring long QT syndrome. Results are consistent in terms of skin sensitization test; all compounds should not develop this effect. However, all compounds may increase hepatotoxicity. The maximum recommended tolerated dose (MRTD) for the whole group should be considered as low. It could be stated that in most tests the results (Table 2) are consistent for the synthesized compounds.

### 2.2. Chemistry

**Scheme 1:**
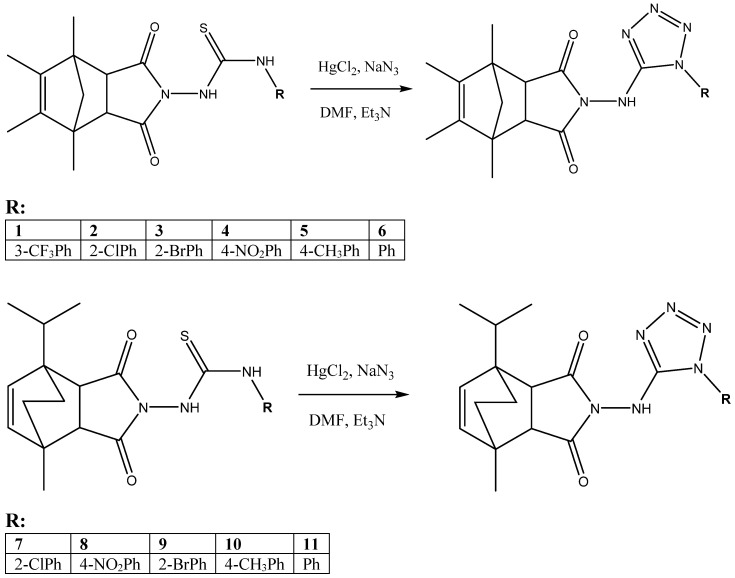
Transition from imide-thiourea substrates to imide-tetrazole hybrids.

Eleven new 1*H*-tetrazol-5-amine derivatives were synthesized according to a well-known procedure [24]. Two types of thiourea derivatives were used. First possessing imide structure of 4,5,6,7-tetramethyl-3a,4,7,7a-tetrahydro-1*H*-4,7-methanoisoindole-1,3(2*H*)-dione and second 4-isopropyl-7-methyl-3a,4,7,7a-tetrahydro-1*H*-4,7-ethanoisoindole-1,3(2*H*)-dione. Procedures for obtaining imides and suitable thiourea derivatives were reported [26,27]. Published papers contain physicochemical and biological evaluation results. The main goal in the current design was to replace the thiourea arrangement with a tetrazole scaffold. Our approach utilizes oxidative desulfurization of thiourea substrates followed by cyclization, using sodium azide as an external nucleophile. Mercury (II) chloride was used as a desulfurization agent. Mechanism of this reaction is known. This structural modification may lead to improvement of antimicrobial activity based on our previous experiments. Both reaction schemes and conditions are depicted below (Scheme 1).

All compounds were obtained in good yields. All eleven compounds were transferred for further physicochemical and biological evaluation. 

### 2.3. Biological Studies

#### 2.3.1. In Vitro Antibacterial Activity Studies

All synthesized compounds were tested in vitro against a set of bacteria, including representative standard Gram-positive and Gram-negative rods. Compounds were screened for their minimal inhibitory concentrations (MICs) [33]. As a result, 4 of 11 examined compounds: **5**, **7**, **9**, and **11** demonstrated moderate antimicrobial activity against standard bacteria strains with MIC values ranging from 32 to 256 μg/mL, while the rest of the tested compounds exhibited high and broad spectrum of activity within the range of 0.1–32 μg/mL (Table 3). It is worth emphasizing that among this group, three compounds (**1**, **2**, **3**) showed excellent antimicrobial profiles, especially against standard Gram-positive *Staphylococci*, within the range of 0.1–3.2 μg/mL and, in general, they were even more effective than the reference Ciprofloxacin. These three compounds were also found to be good inhibitors of the growth of Gram-negative rods: *E. coli* and *P. aeruginosa*, within the range of 0.4–25.6 μg/mL, which indicates that compounds **1**, **2**, **3** are the most promising of all synthesized derivatives.

In the next stage of the experiment, 8 of 11 compounds were selected for a clinical strain activity evaluation against *S. aureus*, *S. epidermidis*, *E. coli*, *K. pneumoniae*, and *P. aeruginosa*. In general, the antimicrobial activity of tested compounds against hospital strains of bacteria is comparable to standard strains (Table 4). MIC values for compounds **4**, **6**, **8**, **9**, **10** range from 2 to 32 μg/mL against Gram-positive strains; however, compound **9** showed better results against all tested clinical microorganisms, including Gram-negative rods. MIC values for compounds **1**, **2**, **3** are within the range of 0.8–3.2 μg/mL. Additionally, all three inhibited the growth of all clinical *Staphylococci* panels: *S. aureus* (T5592; T5591) and *S. epidermidis* (5253; 4243) with MIC values of 0.8 μg/mL. 

In general, the experiment revealed that derivatives of 1,5-disubstituted tetrazole-imides containing 4,5,6,7-tetramethyl moiety exhibit better antimicrobial properties than those with 4-isopropyl-7-methyl imide scaffold. As an exception, compound **10** was more potent than compound **5** against most of the examined standard and clinical bacterial strains. 

Regardless of the imide moiety kind, the presence of the electron-withdrawing nitro substituent in compounds **4** and **8** shows similar antimicrobial properties against the Gram-positive bacterial strains only. What is interesting is that usually nitro substituent induces toxicity of compound and may increase antimicrobial activity of the subjected for evaluation derivative. On the other hand, introduction of the chlorine substituent into *ortho* position to the phenyl ring in compounds **2** and **7** resulted in an antimicrobial activity increase, especially in the case of tetrazole-imides containing the 4,5,6,7-tetramethyl moiety. Exchange of the chlorine into bromine substituent in *ortho* position of the phenyl ring in compound **3** improved the inhibition of bacterial strains growth. Due to the significant electronegativity of the electron-withdrawing trifluoromethyl substituent in the phenyl ring, compound **1** exhibits high and broad antimicrobial activity against all tested strains. Derivatives possessing strong (-CF_3_) and weak (-Br, -Cl) deactivating electron-withdrawing substituents in the benzene ring turned out to have the highest antimicrobial potential of all tested compounds against standard and clinical strains of bacteria. Our previous evaluation of similar compounds showed that in general, electron-donating groups (EDG) attached to phenyl ring or lack of substituent were responsible for a decrease of antibacterial activity.

**Scheme 2:**
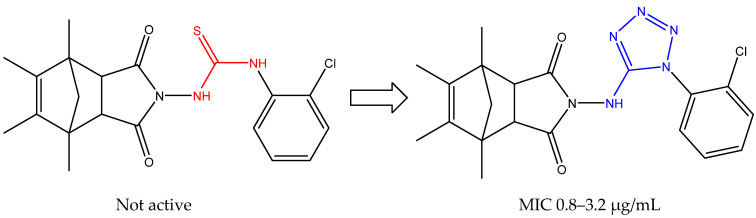
Example of structural modification leading to high activity against bacterial strains.

One of the study’s goals was to determine superior imide moiety (4,5,6,7-tetramethyl in compounds **1**–**6** or 4-isopropyl-7-methyl in compounds **7**–**11**). Containing 4,5,6,7-tetramethyl moiety, derivatives **1**, **2**, and **3** showed significant antimicrobial results. In the second group of synthesized compounds, we observed no similar leading structures. Finally, the primary outcome of the study was achieved. Transition from substrate imide-thioureas [29] to imide-tetrazole derivatives resulted in a spectacular increase of antimicrobial properties. Most of the substrates were inactive against standard bacteria strains; for only four (out of twenty-six derivatives), good activity was established. Such synthetic transition followed by antimicrobial activity improvement is depicted below (Scheme 2).

Lead compounds **1**, **2**, **3** were transferred for further testing to establish mechanism of antimicrobial action.

#### 2.3.2. Inhibition of Catalytic Activities of *S. aureus* Topoisomerases

Topoisomerase IV is a bacterial type topoisomerase that is essential for proper chromosome segregation. It is the primary target of second-generation fluoroquinolones, such as Ciprofloxacin and Levofloxacin. Another type of bacterial type II topoisomerase is DNA gyrase. In general, it is supposed that in Gram-positive bacteria species, topoisomerase IV rather than DNA gyrase appears to be the primary target of most quinolone-based antibiotics. In this work, the influence of tetrazole derivatives was tested for both topoisomerase IV and DNA gyrase.

**Figure 1:**
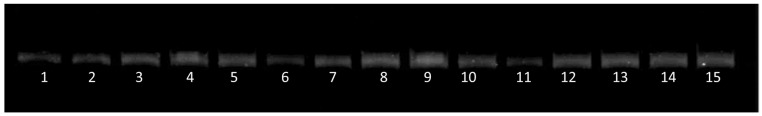
The effect of tested compounds on *S. aureus* topoisomerase IV activity. Decreasing amounts of **1**, **2**, or **3** were incubated with 200 ng kinetoplast DNA and run-on agarose gel. Lane: 1–5: compound **3** at concentrations 64, 32, 8, 2, 0.5 μg/mL, respectively. Lane: 6–10: compound **2** at concentrations 64, 32, 8, 2, 0.5 μg/mL, respectively. Lane: 11–15: compound **1** at concentrations 64, 32, 8, 2, 0.5 μg/mL, respectively.

**Figure 2:**
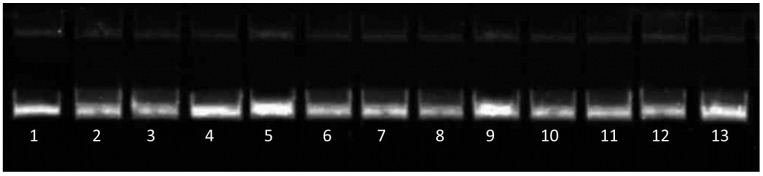
The influence of tested compounds on *S. aureus* DNA gyrase activity. Decreasing amounts of **1**, **2**, or **3** were incubated with supercoiled pBR322 plasmid DNA (500 ng) and run-on agarose gel. Lane 1: *S. aureus* DNA gyrase assay with DMSO (control). Lane: 2–5: compound **3** at concentrations 64, 32, 8, 2 μg/mL, respectively. Lane: 6–9: compound **2** at concentrations 64, 32, 8, 2 μg/mL, respectively. Lane: 10–13: compound **1** at concentrations 64, 32, 8, 2 μg/mL, respectively.

Selected compounds with the highest antimicrobial activity **1**, **2**, **3** were examined in topoisomerase IV decatenation assay (Figure 1) and DNA gyrase supercoiling assay (Figure 2). 

The main outcome of the study is that compounds were able to inhibit the activity of bacterial gyrase and topoisomerases IV from *S. aureus*. The half minimal inhibitory concentration (IC_50_) results (see Table 5) for bacterial topoisomerases were higher than reference Ciprofloxacin and Moxifloxacin. 

Moreover, the presented IC_50_ values suggest that there is a higher affinity towards the DNA gyrase of synthesized derivatives. What needs to be stated is that IC_50_ values obtained in in vitro study showed indecisive levels of affinity towards topoisomerases. There are significant structural differences between synthesized compounds and fluoroquinolones such as reference Ciprofloxacin and Moxifloxacin. There is a possibility that the antimicrobial mechanism of action can be dual (two preferred binding places of topoisomerase) or related to another antibacterial mode of action. 

### 2.4. Molecular Docking

To gain some insight into the possible binding modes, we conducted molecular docking of the studied compounds **1**, **2**, and **3** (the docking procedure is described in the Methods section). 

The docking was carried out in two stages. First to the binding site targeted by fluoroquinolones (e.g., Ciprofloxacin, see our previous works [34]. The fluoroquinolones bind to the DNA–enzyme complex and stabilize it in the cleaved DNA state. The molecular docking to the fluoroquinolone binding site (using the crystal structure of the DNA gyrase in complex with DNA, PDB ID: 5BTC) showed that the narrow binding cleft is not well-suited to the docked ligands. Namely, the cleft requires a planar ligand structure, such as Ciprofloxacin, rather than non-flat **1**–**3** compounds. 

**Figure 3:**
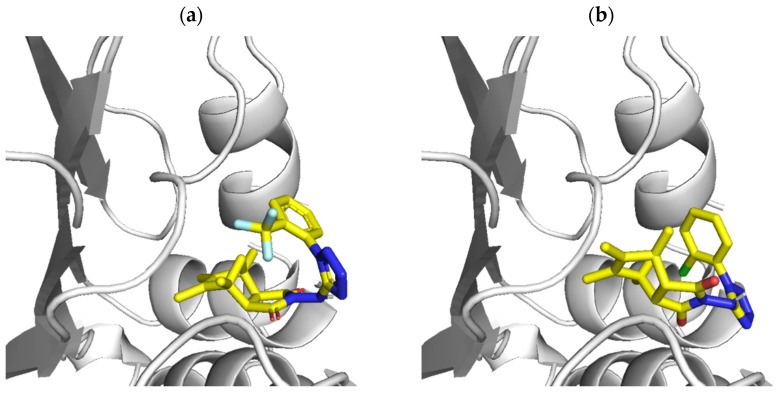

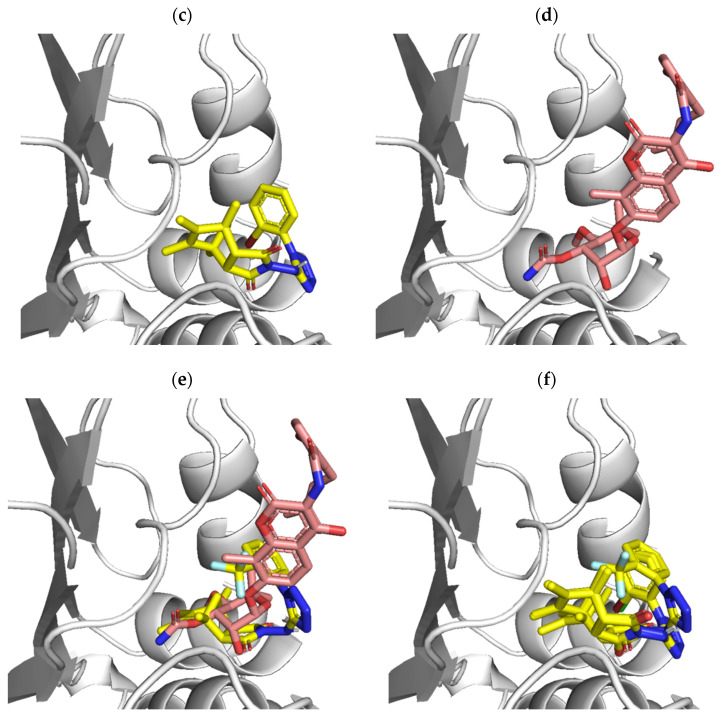
Molecular docking results and comparison to the Novobiocin-gyrase complex. (**a**) **1**, (**b**) **2**, (**c**) **3**, (**d**) Novobiocin (PDB ID: 4URO), (**e**) comparison of **1** and Novobiocin, (**f**) comparison of **1**, **2**, and **3** ligands.

In addition to the fluoroquinolone inhibitor, there are also ATP-competitive inhibitors that target the B subunit of DNA gyrase with Novobiocin as the best-studied representative. Thus, in the second stage, we targeted the ATP-binding site. In docking, *Staphylococcus aureus* DNA Gyrase subunit B (crystal structure in complex with Novobiocin; PDB ID: 4URO) has been used. The docking results show that the docked **1**–**3** compounds occupy the ATP binding site with favorable binding energy (with ligand **1** showing a slightly higher preference for binding than other ligands, see Table 6 and Figure 3). In comparison to the Novobiocin-binding mode, the ligands mainly bind to the gyrase site that is responsible for interactions with the sugar Novobiocin moiety. In summary, the presented docking results indicate the possibility of binding to the ATP site; however, this binding mode requires further experimental confirmation.

## 3. Materials and Methods

### 3.1. Apparatus, Materials, and Analysis

The reagents were supplied from Alfa Aesar (Haverhill, MA, USA) or Sigma Aldrich (Saint Louis, MO, USA). Organic solvents (acetonitrile, DMF, chloroform, and methanol) were supplied from POCh (Polskie Odczynniki Chemiczne, Gliwice, Poland). All chemicals were of analytical grade. Before use, dried DMF and acetonitrile were kept in crown cap bottles over anhydrous phosphorus pentoxide (Carl Roth, Karlsruhe, Germany).

The NMR spectra were recorded on a Bruker AVANCE DMX400 (Bruker, Billerica, MA, USA) spectrometer, operating at 500 or 300 MHz (^1^H NMR) and 125 or 75 MHz (^13^C NMR). The chemical shift values are expressed in ppm relative to TMS as an internal standard. Mass spectral ESI measurements were carried out on Waters ZQ Micro-mass instruments (Waters, Milford, MA, USA) with a quadrupole mass analyzer. The spectra were performed in the positive ion mode at a declustering potential of 40–60 V. The sample was previously separated on a UPLC column (C18) using the UPLC ACQUITYTM system by Waters connected with a DPA detector. Flash chromatography was performed on Merck silica gel 60 (200–400 mesh) using chloroform/methanol (19:1 vol) mixture as eluent. Analytical TLC was carried out on silica gel F254 (Merck) plates (0.25 mm thickness). 

### 3.2. Imide-Tetrazole Hybrids Preparation: Derivatives of 3a,4,7,7a-Tetrahydro-1H-4,7-methanoisoindole-1,3(2H)-dione and 4-Isopropyl-7-methyl-3a,4,7,7a-tetrahydro-1H-4,7-ethanoisoindole-1,3(2H)-dione

Triethylamine (503 μL, 3.75 mmol, 1–3 drops) was added to a suspension of a corresponding thiourea substrate (1.25 mmol), sodium azide (244 mg, 3.75 mmol), and mercuric chloride (373 mg, 1.38 mmol) in 20 mL of dry DMF. The resulting suspension was stirred for 6 h at room temperature or until TLC indicated complete consumption of starting material. The suspension was filtered through a pad of celite, washing with CH_2_Cl_2_. The filtrate was diluted with water, and extracted with 3 × 15 mL of CH_2_Cl_2_. The combined organics were dried over MgSO_4_, filtered, and concentrated under reduced pressure. The resulting residue was purified by silica gel chromatography.

#### 3.2.1. 4,5,6,7-Tetramethyl-2-((1-(3-(trifluoromethyl)phenyl)-1H-tetrazol-5-yl)amino)-3a,4,7,7a-tetrahydro-1H-4,7-methanoisoindole-1,3(2H)-dione (**1**)

Yield 68%. ^1^H NMR (300 MHz, DMSO-d_6_): δ 1.25 (d, *J* = 8.1 Hz, 1H, CH_2_), 1.37 (s, 6H, 2xCH_3_), 1.47 (d, *J* = 8.4 Hz, 1H, CH_2_), 1.50 (s, 6H, 2xCH_3_), 3.05 (s, 2H, 2xCH), 7.38 (d, *J* = 7.8 Hz, 1H, ArH), 7.51 (t, *J* = 7.9 Hz, 1H, ArH), 7.66 (d, *J* = 8.1 Hz, 1H, ArH), 7.90 (s, 1H, ArH), 10.15 (s, 1H, NH). ^13^C NMR (75 MHz, DMSO-d_6_): δ 11.1 (2xC), 16.5 (2xC), 50.7 (2xC), 54.2 (2xC), 62.6, 118.4, 119.7, 124.1 (q, *J*_1_ = 272.5 Hz), 125.9, 128.7 (q, *J*_2_ = 31.0 Hz), 129.4, 136.8 (2xC), 140.6, 153.6, 173.6 (2xC=O). HRMS (ESI) calc. for C_21_H_21_F_3_N_6_O_2_ [M-H]^−^: 445.4342 found: 445.4339.

#### 3.2.2. 2-((1-(2-Chlorophenyl)-1H-tetrazol-5-yl)amino)-4,5,6,7-tetramethyl-3a,4,7,7a-tetrahydro-1H-4,7-methanoisoindole-1,3(2H)-dione (**2**)

Yield 91%. ^1^H NMR (300 MHz, DMSO-d_6_): δ 1.25 (d, *J* = 8.1 Hz, 1H, CH_2_), 1.38 (s, 6H, 2xCH_3_), 1.47 (d, *J* = 8.4 Hz, 1H, CH_2_), 1.51 (s, 6H, 2xCH_3_), 3.06 (s, 2H, 2xCH), 7.59–7.74 (m, 3H, ArH), 7.79–7.82 (m, 1H, ArH), 10.21 (s, 1H, NH). ^13^C NMR (75 MHz, DMSO-d_6_): δ 11.1 (2xC), 16.6 (2xC), 50.7 (2xC), 54.2 (2xC), 62.6, 128.7, 129.7, 129.7, 130.7, 131.2, 133.0, 136.8 (2xC), 153.7, 173.6 (2xC=O). HRMS (ESI) calc. for C_20_H_21_ClN_6_O_2_ [M-H]^−^: 412.8780 found: 412.8782.

#### 3.2.3. 2-((1-(2-Bromophenyl)-1H-tetrazol-5-yl)amino)-4,5,6,7-tetramethyl-3a,4,7,7a-tetrahydro-1H-4,7-methanoisoindole-1,3(2H)-dione (**3**)

Yield 75%. ^1^H NMR (300 MHz, DMSO-d_6_): δ 1.25 (d, *J* = 8.1 Hz, 1H, CH_2_), 1.37 (s, 6H, 2xCH_3_), 1.47 (d, *J* = 8.4 Hz, 1H, CH_2_), 1.50 (s, 6H, 2xCH_3_), 3.05 (s, 2H, 2xCH), 7.61–7.67 (m, 3H, ArH), 7.92–7.95 (m, 1H, ArH), 10.15 (s, 1H, NH). ^13^C NMR (75 MHz, DMSO-d_6_): δ 11.1 (2xC), 16.5 (2xC), 50.7 (2xC), 54.2 (2xC), 62.6, 121.3, 129.3, 129.8, 131.3, 133.1, 133.8, 136.8 (2xC), 153.6, 173.6 (2xC=O). HRMS (ESI) calc. for C_20_H_21_BrN_6_O_2_ [M-H]^−^: 457.3320 found: 457.3323.

#### 3.2.4. 4,5,6,7-Tetramethyl-2-((1-(4-nitrophenyl)-1H-tetrazol-5-yl)amino)-3a,4,7,7a-tetrahydro-1H-4,7-methanoisoindole-1,3(2H)-dione (**4**)

Yield 71%. ^1^H NMR (300 MHz, DMSO-d_6_): δ 1.33 (d, *J* = 8.4 Hz, 1H, CH_2_), 1.45 (s, 6H, 2xCH_3_), 1.53 (s, 6H, 2xCH_3_), 1.67 (d, *J* = 8.4 Hz, 1H, CH_2_), 3.48 (s, 2H, 2xCH), 7.81–7.86 (m, 2H, ArH), 8.30–8.35 (m, 2H, ArH), 10.74 (s, 1H, NH). ^13^C NMR (75 MHz, DMSO-d_6_): δ 10.6 (2xC), 16.2 (2xC), 51.7 (2xC), 55.4 (2xC), 62.0, 117.8 (2xC), 125.5 (2xC), 137.2 (2xC), 142.1, 144.1, 150.7, 171.5 (2xC=O). HRMS (ESI) calc. for C_20_H_21_N_7_O_4_ [M-H]^−^: 422.4330 found: 422.4327.

#### 3.2.5. 4,5,6,7-Tetramethyl-2-((1-(p-tolyl)-1H-tetrazol-5-yl)amino)-3a,4,7,7a-tetrahydro-1H-4,7-methanoisoindole-1,3(2H)-dione (**5**)

Yield 82%. ^1^H NMR (300 MHz, DMSO-d_6_): δ 1.24 (d, *J* = 8.1 Hz, 1H, CH_2_), 1.38 (s, 6H, 2xCH_3_), 1.48 (d, *J* = 8.4 Hz, 1H, CH_2_), 1.51 (s, 6H, 2xCH_3_), 2.42 (s, 3H, CH_3_), 3.08 (s, 2H, 2xCH), 7.45 (s, 4H, ArH), 10.07 (s, 1H, NH). ^13^C NMR (75 MHz, DMSO-d_6_): δ 11.1 (2xC), 16.6 (2xC), 20.8, 50.7 (2xC), 54.2 (2xC), 62.6, 124.6 (2xC), 130.0, 130.4 (2xC), 136.8 (2xC), 140.2, 153.7, 173.6 (2xC=O). HRMS (ESI) calc. for C_21_H_24_N_6_O_2_ [M-H]^−^: 391.4630 found: 391.4632.

#### 3.2.6. 4,5,6,7-Tetramethyl-2-((1-phenyl-1H-tetrazol-5-yl)amino)-3a,4,7,7a-tetrahydro-1H-4,7-methanoisoindole-1,3(2H)-dione (**6**)

Yield 93%. ^1^H NMR (300 MHz, DMSO-d_6_): δ 1.25 (d, *J* = 8.1 Hz, 1H, CH_2_), 1.37 (s, 6H, 2xCH_3_), 1.47 (d, *J* = 8.4 Hz, 1H, CH_2_), 1.50 (s, 6H, 2xCH_3_), 3.06 (s, 2H, 2xCH), 7.55–7.58 (m, 2H, ArH), 7.62–7.69 (m, 3H, ArH), 10.16 (s, 1H, NH). ^13^C NMR (75 MHz, DMSO-d_6_): δ 11.1 (2xC), 16.6 (2xC), 50.7 (2xC), 54.2 (2xC), 62.6, 124.7 (2xC), 130.0 (2xC), 130.3, 132.5, 136.8 (2xC), 153.6, 173.6 (2xC=O). HRMS (ESI) calc. for C_20_H_22_N_6_O_2_ [M-H]^−^: 377.4360 found: 377.4358.

#### 3.2.7. 2-((1-(2-Chlorophenyl)-1H-tetrazol-5-yl)amino)-4-isopropyl-7-methyl-3a,4,7,7a-tetrahydro-1H-4,7-ethanoisoindole-1,3(2H)-dione (**7**)

Yield 81%. ^1^H NMR (300 MHz, DMSO-d_6_): δ 0.93 (d, *J* = 6.9 Hz, 3H, CH_3_), 1.03 (d, *J* = 6.9 Hz, 3H, CH_3_), 1.14–1.25 (m, 2H, CH_2_), 1.38 (s, 3H, CH_3_), 1.44–1.58 (m, 2H, CH_2_), 2.39–2.48 (m, 1H, CH), 2.80 (d, *J* = 8.1 Hz, 1H, CH), 3.10 (d, *J* = 8.1 Hz, 1H, CH), 5.88 (d, *J* = 8.4 Hz, 1H, CH), 5.96 (d, *J* = 8.4 Hz, 1H, CH), 7.56–7.73 (m, 3H, ArH), 7.78–7.82 (m, 1H, ArH), 10.20 (s, 1H, NH). ^13^C NMR (75 MHz, DMSO-d_6_): δ 16.8, 18.2, 22.3, 22.4, 29.3, 33.5, 36.3, 43.1, 44.2, 47.8, 128.7, 129.7, 129.7, 130.7, 131.2, 133.0, 135.3, 136.3, 154.5, 173.1, 173.6.

HRMS (ESI) calc. for C_21_H_23_ClN_6_O_2_ [M-H]^−^: 425.9050 found: 425.9049.

#### 3.2.8. 4-Isopropyl-7-methyl-2-((1-(4-nitrophenyl)-1H-tetrazol-5-yl)amino)-3a,4,7,7a-tetrahydro-1H-4,7-ethanoisoindole-1,3(2H)-dione (**8**)

Yield 74%. ^1^H NMR (500 MHz, DMSO-d_6_): δ 0.95 (d, *J* = 6.5 Hz, 3H, CH_3_), 1.07 (d, *J* = 7.0 Hz, 3H, CH_3_), 1.17–1.24 (m, 2H, CH_2_), 1.43 (s, 3H, CH_3_), 1.51–1.62 (m, 2H, CH_2_), 2.41–2.45 (m, 1H, CH), 3.15 (d, *J* = 8.0 Hz, 1H, CH), 3.39 (d, *J* = 8.0 Hz, 1H, CH), 6.05 (d, *J* = 8.5 Hz, 1H, CH), 6.14 (d, *J* = 8.5 Hz, 1H, CH), 7.86 (d, *J* = 9.0 Hz, 2H, ArH), 8.31 (d, *J* = 9.5 Hz, 2H, ArH), 10.76 (s, 1H, NH). ^13^C NMR (125 MHz, DMSO-d_6_): δ 16.7, 18.1, 22.1, 22.3, 29.3, 33.1, 36.7, 43.5, 45.4, 48.8, 117.9 (2xC), 125.3, 125.5, 135.5, 136.6, 142.2, 144.1, 150.5, 170.9, 171.4. HRMS (ESI) calc. for C_21_H_23_N_7_O_4_ [M-H]^−^: 436.4600 found: 436.4602.

#### 3.2.9. 2-((1-(2-Bromophenyl)-1H-tetrazol-5-yl)amino)-4-isopropyl-7-methyl-3a,4,7,7a-tetrahydro-1H-4,7-ethanoisoindole-1,3(2H)-dione (**9**)

Yield 81%. ^1^H NMR (300 MHz, DMSO-d_6_): δ 0.93 (d, *J* = 6.9 Hz, 3H, CH_3_), 1.03 (d, *J* = 6.6 Hz, 3H, CH_3_), 1.14–1.25 (m, 2H, CH_2_), 1.38 (s, 3H, CH_3_), 1.44–1.58 (m, 2H, CH_2_), 2.39–2.48 (m, 1H, CH), 2.80 (d, *J* = 8.1 Hz, 1H, CH), 3.10 (d, *J* = 8.1 Hz, 1H, CH), 5.88 (d, *J* = 8.4 Hz, 1H, CH), 5.96 (d, *J* = 8.4 Hz, 1H, CH), 7.61–7.67 (m, 3H, ArH), 7.92–7.95 (m, 1H, ArH), 10.16 (s, 1H, NH). ^13^C NMR (75 MHz, DMSO-d_6_): δ 16.8, 18.2, 22.3, 22.4, 29.3, 33.5, 36.3, 43.1, 44.2, 47.8, 121.4, 129.3, 129.8, 131.3, 133.1, 133.8, 135.3, 136.3, 154.3, 173.1, 173.5.

HRMS (ESI) calc. for C_20_H_21_BrN_6_O_2_ [M-H]^−^: 456.3320 found: 456.3318.

#### 3.2.10. 4-Isopropyl-7-methyl-2-((1-(p-tolyl)-1H-tetrazol-5-yl)amino)-3a,4,7,7a-tetrahydro-1H-4,7-ethanoisoindole-1,3(2H)-dione (**10**)

Yield 90%. ^1^H NMR (300 MHz, DMSO-d_6_): δ 0.93 (d, *J* = 6.9 Hz, 3H, CH_3_), 1.03 (d, *J* = 6.6 Hz, 3H, CH_3_), 1.11–1.25 (m, 2H, CH_2_), 1.38 (s, 3H, CH_3_), 1.48–1.58 (m, 2H, CH_2_), 2.39–2.46 (m, 4H, CH_3_ and CH), 2.90 (d, *J* = 8.1 Hz, 1H, CH), 3.09 (d, *J* = 7.8 Hz, 1H, CH), 5.87 (d, *J* = 8.4 Hz, 1H, CH), 5.95 (d, *J* = 8.7 Hz, 1H, CH), 7.45 (s, 4H, ArH), 10.12 (s, 1H, NH). ^13^C NMR (75 MHz, DMSO-d_6_): δ 16.7, 18.2, 22.3, 22.4, 29.3, 33.5, 36.3, 43.1, 44.3, 47.8, 124.5 (2xC), 129.9, 130.3 (2xC), 135.2, 136.2, 140.1, 153.7, 173.3, 173.7. HRMS (ESI) calc. for C_22_H_26_N_6_O_2_ [M-H]^−^: 405.4900 found: 405.4903.

#### 3.2.11. 4-Isopropyl-7-methyl-2-((1-phenyl-1H-tetrazol-5-yl)amino)-3a,4,7,7a-tetrahydro-1H-4,7-ethanoisoindole-1,3(2H)-dione (**11**)

Yield 94%. ^1^H NMR (300 MHz, DMSO-d_6_): δ 0.93 (d, *J* = 6.0 Hz, 3H, CH_3_), 1.03 (d, *J* = 6.3 Hz, 3H, CH_3_), 1.15–1.25 (m, 2H, CH_2_), 1.39 (s, 3H, CH_3_), 1.47–1.58 (m, 2H, CH_2_), 2.40–2.46 (m, 1H, CH), 2.79 (d, *J* = 7.8 Hz, 1H, CH), 3.09 (d, *J* = 7.5 Hz, 1H, CH), 5.88 (d, *J* = 8.4 Hz, 1H, CH), 5.96 (d, *J* = 8.1 Hz, 1H, CH), 7.57–7.69 (m, 5H, ArH), 10.21 (s, 1H, NH). ^13^C NMR (75 MHz, DMSO-d_6_): δ 16.8, 18.2, 22.3, 22.4, 29.3, 33.4, 36.3, 43.1, 44.3, 47.8, 124.6 (2xC), 130.0 (2xC), 130.2, 132.4, 135.2, 136.2, 153.7, 173.3, 173.7. HRMS (ESI) calc. for C_21_H_24_N_6_O_2_ [M-H]^−^: 391.4630 found: 391.4628.

### 3.3. Biological Assays

The antimicrobial assays were conducted using reference strains of bacteria derived from international microbe collections: American Type Culture Collection (ATCC) and National Collection of Type Culture (NCTC). The following standard strains of bacteria were used: Gram-positive—*Staphylococcus aureus* NCTC 4163, *Staphylococcus aureus* ATCC 25923, *Staphylococcus aureus* ATCC 6538, *Staphylococcus aureus* ATCC 29213, *Staphylococcus epidermidis* ATCC 12228, *Staphylococcus epidermidis* ATCC 35984, Gram-negative: *Escherichia coli* ATCC 25922, *Pseudomonas aeruginosa* ATCC 15442. Clinical strains of bacteria used in this study: Gram-positive: *Staphylococcus epidermidis* 5253, *Staphylococcus epidermidis* 4243, *Staphylococcus aureus* T5592, *Staphylococcus aureus* T5591 and Gram-negative: *Escherichia coli* 520, *Escherichia coli* 600, *Klebsiella pneumoniae* 510 and *Pseudomonas aeruginosa* 659 were obtained from the collection of the Department of Pharmaceutical Microbiology, Medical University of Warsaw, Poland and they were isolated from different biological materials taken from the patients hospitalized in the Warsaw Medical University hospitals. Antimicrobial activity was examined by the minimal inhibitory concentration (MIC) method under standard procedures provided by CLSI with some modifications. MIC was determined by the two-fold serial broth microdilution method in 96-well microtitration plates using Mueller–Hinton II broth medium (Becton Dickinson, Franklin Lakes, NJ, USA). The final inoculum of all studied bacteria was 10^6^ CFU/mL (colony forming unit per milliliter). The stock solution of tested compounds was prepared in dimethyl sulfoxide (DMSO) and diluted to a maximum of 1% of solvent content with a sterile medium. The MIC value recorded is defined as the lowest concentration of the tested antimicrobial agents (expressed in µg/mL) that inhibit the visible growth of the microorganism after 19 h of incubation at 35 °C.

Description related to conducted biological studies including cell culture, suitable conditions, and methodology was presented in our previous paper [34].

### 3.4. Molecular Docking Studies

The molecular docking procedure was as follows. First, the ligand structures were generated using the Automated Topology Builder server (ATB version 2.2) [35]. Docking calculations and data analysis were performed using AutoDock4 (v. 4.2) and AutoDockTools4 [36]. For each receptor-ligand complex, 1000 independent docking cycles were performed, resulting in 1000 conformers with the lowest binding energy. Next, structural clustering was used to identify the most preferred binding modes (with RMSD cut-off of 3 Å). Finally, the central structure of the largest cluster was selected as the final ligand-docked structure for each complex.

## 4. Conclusions

The transition presented here from imide-thiourea substrates, thoroughly studied in our previous works [22,23,24,25], to appropriate novel imide-tetrazole products was successfully performed in the course of a single reaction. All synthesized compounds were tested in vitro against a set of bacteria, including representative standard Gram-positive and Gram-negative rods. Transition from substrate imide-thioureas to imide-tetrazoles increased antimicrobial properties of obtained derivatives. Compounds **1**, **2**, and **3** possessing strong (-CF_3_) and weak (-Br, -Cl) deactivating electron-withdrawing substituents in the benzene ring turned out to have the highest antimicrobial potential of all tested compounds against standard and clinical strains of bacteria. Lead compounds **1**, **2**, **3** were transferred for further testing to establish the mechanism of antimicrobial action. Those were examined in topoisomerase IV decatenation assay and DNA gyrase supercoiling assay. Obtained results suggest that there is a higher affinity towards the DNA gyrase of synthesized derivatives. We also conducted molecular docking of the studied compounds to gain some insight into the possible binding modes. The molecular docking to the fluoroquinolone binding site showed that the narrow binding cleft is not well-suited to the docked ligands. In the second stage, we targeted the ATP-binding site of the B subunit of DNA gyrase with Novobiocin as the best-studied representative. In comparison to the Novobiocin-binding mode, the ligands mainly bind to the gyrase site that is responsible for interactions with the sugar Novobiocin moiety. 

Summarizing, the presented transition from substrate imide-thioureas to imide-tetrazole derivatives resulted in significant increase of antimicrobial properties. The compounds **1**–**3** proposed here provide a promising basis for further exploration towards novel antimicrobial drug candidates. 

## Figures and Tables

**Table 1:** Examples of several of the *S. aureus* mechanisms of resistance to selected antibiotics.

Antibiotic	Resistance Gene(s)	Gene Product(s)	Mechanism(s) of Resistance	Location(s)
β-Lactams	(1) *blaZ* (2) *mecA*	(1) β-Lactamase (2) PBP2a	(1) Enzymatic hydrolysis of β-lactam nucleus (2) Reduced affinity for PBP	(1) Pl:Tn (2) C:SCC*mec*
Glycopeptides	(1) Unknown (VISA) (2) VanH_A_	(1) Altered peptidoglycan (2) D-Ala-D-Lac	(1) Trapping of vancomycin in the cell wall (2) Synthesis of dipeptide with reduced affinity for vancomycin	(1) C (2) Pl:Tn
Quinolones	(1) *parC* (2) *gyrA* or *gyrB*	(1) ParC (or GrlA) component of topoisomerase IV (2) GyrA or GyrB components of gyrase	(1, 2) Mutations in the QRDR region, reducing affinity of enzyme-DNA complex for quinolones	(1) C (2) C
Aminoglycosides (e.g., gentamicin) Trimethoprim-sulfamethoxazole (TMP-SMZ)	Aminoglycoside-modifying enzymes (e.g., *aac*, *aph*) (1) Sulfonamide: *sulA* (2) *TMP: dfrB*	Acetyltransferase, phosphotransferase (1) Dihydropteroate synthase (2) Dihydrofolate reductase (DHFR)	Acetylating and/or phosphorylating enzymes modify aminoglycosides (1) Overproduction of *p*-aminobenzoic acid by enzyme (2) Reduced affinity for DHFR	Pl, Pl:Tn (1) C (2) C
Oxazolidinones	*rrn*	23S rRNA	Mutations in domain V of 23S rRNA component of the 50S ribosome. Interferes with ribosomal binding	C
Quinupristin-dalfopristin (Q-D)	(1) Q: *ermA*, *ermB*, *ermC* (2) D: *vat*, *vatB*	(1) Ribosomal methylases (2) Acetyltransferases	(1) Reduce binding to the 23S ribosomal subunit (2) Enzymatic modification of dalfopristin	(1) Pl, C (2) Pl

Pl, plasmid; C, chromosome; Tn, transposon; QRDR, quinolone resistance-determining region.

**Table 2:** The toxicological parameters of synthesized imide-tetrazole derivatives estimated using pkCSM software.

Toxicological Test	Unit	1	2	3	4	5	6	7	8	9	10	11
AMES toxicity	(Yes/No)	No	No	No	Yes	No	No	No	Yes	No	No	No
Max. tolerated dose (human)	(log mg/kg/day)	0.309	0.361	0.355	−0.002	0.402	0.402	0.267	−0.055	0.272	−0.685	0.297
hERG I inhibitor	(Yes/No)	No	No	No	No	No	No	No	No	No	No	No
hERG II inhibitor	(Yes/No)	Yes	Yes	Yes	Yes	Yes	Yes	Yes	Yes	Yes	No	Yes
Oral Rat Acute Toxicity (LD50)	(mol/kg)	2.749	2.589	2598	2.483	2.417	2.47	2.636	2.542	2.558	1.834	2.517
Oral Rat Chronic Toxicity (LOAEL)	(log mg/kg_bw/day)	1.025	1.218	1.207	1.345	1.487	1.42	1.213	1.337	1.245	1.944	1.416
Hepatotoxicity	(Yes/No)	Yes	Yes	Yes	Yes	Yes	Yes	Yes	Yes	Yes	Yes	Yes
Skin Sensitization	(Yes/No)	No	No	No	No	No	No	No	No	No	No	No
*T. Pyriformis* toxicity	(log ug/L)	0.294	0.297	0.297	0.287	0.301	0.3	0.29	0.286	0.291	0.29	0.291
Minnow toxicity	(log mM)	−0.453	−0.621	−0.767	−1.597	−0.913	−0.306	−0.95	−1.914	−1.606	−2.571	−0.635

**Table 3:** Activity of compounds against standard bacteria strains, expressed by minimal inhibitory concentrations (µg/mL).

Strain	Compound (µg/mL)											
	1	2	3	4	5	6	7	8	9	10	11	Ciprofloxacin (µg/mL)
*S. aureus* NCTC 4163	0.2	0.4	0.1	16	>256	2	32	8	64	4	128	0.125
*S. aureus* ATCC 25923	0.2	0.4	0.1	8	>256	2	32	8	64	4	128	0.5
*S. aureus* ATCC 6538	0.2	0.4	0.2	32	>256	2	32	8	64	4	128	0.25
*S. aureus* ATCC 29213	0.2	0.8	0.2	16	>256	4	64	8	64	8	128	0.25
*S. epidermidis* ATCC 12228	3.2	3.2	2	32	>256	16	128	16	128	32	256	0.25
*S. epidermidis* ATCC 35984	0.2	0.4	0.2	32	>256	4	32	16	64	8	128	0.125
*E. coli* ATCC 25922	0.4	1.6	0.4	256	>256	8	128	>256	128	16	>256	0.015
*P. aeruginosa* ATCC 15442	12.8	25.6	16	>256	>256	>256	>256	>256	>256	>256	>256	0.125

**Table 4:** Activity of the selected derivatives against clinical isolates, expressed by minimal inhibitory concentrations (µg/mL).

Strain	Compound (µg/mL)								
	1	2	3	4	6	8	9	10	Ciprofloxacin (µg/mL)
*S. epidermidis* 5253	0.8	0.8	0.8	16	4	16	4	16	0.25
*S. epidermidis* 4243	0.8	0.8	0.8	32	4	16	2	8	0.13
*S. aureus* T5592	0.8	0.8	0.8	16	4	16	4	16	0.25
*S. aureus* T5591	0.8	0.8	0.8	32	4	16	4	16	0.25
*E. coli* 520	3.2	3.2	1.6	256	8	>256	8	32	0.06
*E. coli* 600	3.2	1.6	1.6	256	8	>256	8	32	0.03
*K. pneumoniae* 510	3.2	3.2	3.2	256	16	>256	16	32	4
*P. aeruginosa* 659	1.6	1.6	1.6	256	16	>256	16	32	0.5

**Table 5:** The affinity of selected compounds towards bacterial type II topoisomerases, expressed as IC_50_ ± SEM (µg/mL).

Compounds	IC_50_ ± S.E.M. for Topoisomerase IV (µg/mL)	IC_50_ ± S.E.M. for DNA Gyrase (µg/mL)
**Ciprofloxacin**	1.70 ± 0.15	3.55 ± 0.13
**Moxifloxacin**	0.9 ± 0.1	12.05 ± 0.50
**1**	63.2 ± 4.2	32.06 ± 4.04
**2**	58.4 ± 3.5	37.52 ± 9.72
**3**	61.2 ± 2.1	45.43 ± 3.54

**Table 6:** Docking results characterized by number of structures in the largest cluster and binding energy.

Ligand (Compound)	Number of Structures in the Largest Cluster	Binding Energy
**3**	598	−5.09 [kcal/mol]
**2**	609	−4.97 [kcal/mol]
**1**	818	−5.58 [kcal/mol]

## Data Availability

Not applicable.
